# Proteolytic Activity of Commercial Thermophilic Starter Cultures and Changes in Protein Fractions and Free Amino Acids in Organic and Conventional Fermented Milk

**DOI:** 10.1002/fsn3.72199

**Published:** 2026-08-02

**Authors:** Stefanija Steinweg, Jelena Zagorska, Kristine Majore, Vitalijs Komasilovs, Vitalijs Radenkovs, Inga Ciprovica

**Affiliations:** ^1^ Food Institute Latvia University of Life Sciences and Technologies Jelgava Latvia; ^2^ Institute of Computer Systems and Data Science Latvia University of Life Sciences and Technologies Jelgava Latvia; ^3^ Division of Smart Technologies Latvia University of Life Sciences and Technologies Jelgava Latvia; ^4^ Institute of Horticulture Dobele Latvia

**Keywords:** conventional, fermented milk, free amino acids, organic, protein fractions, protein hydrolysis

## Abstract

Starter culture (SC) composition and milk matrix characteristics shape fermentation and proteolysis‐related changes in fermented milk (FM). This study evaluated the effects of milk origin (organic (ORG) and conventional (CNV)), commercial thermophilic SCs composition, and 7‐day refrigerated storage on bacterial viability, proteolytic activity, protein fraction (PF) hydrolysis, and free amino acid (FAA) accumulation. ORG and CNV pasteurized milk collected over 1 year (twice outdoor and indoor period) was fermented with commercial starters containing *Lactobacillus delbrueckii* subsp. *bulgaricus*, *Lactobacillus helveticus* , and *Streptococcus thermophilus* . Fermentation reduced pH to 4.60–4.63 and increased proteolytic activity from 68.50–72.00 AU in pasteurized milk to 140.83–169.67 AU in FM. After storage, proteolytic activity further increased to 187.33–234.00 AU, with the highest value in CNV/TCC‐20. Nonprotein nitrogen compounds increased from 0.29 g 100 g^−1^ to 0.33–0.42 g 100 g^−1^ after storage. Total identified PFs content decreased by 8.0%–24.2% after fermentation, with the greatest reduction in CNV/YFL902, and TCC‐20 samples showed further decreases of approximately 22%–24% during storage. Total FAAs content increased by 6.7%–30.4% after fermentation, but after storage in all samples, except YFL902, decreased by 18.1%–46.8%. Overall, SC composition and storage time dominated proteolysis‐related changes, while milk origin mainly modulated selected storage responses.

## Graphical abstract

This study evaluated the impact of milk origin (organic vs. conventional) and starter culture composition on LAB proteolytic activity, and related changes in non‐protein nitrogen compounds in fermented milk. Results provide insight into protein hydrolysis, individual protein fractions and free amino acids in organic and conventional milk after fermentation and storage.

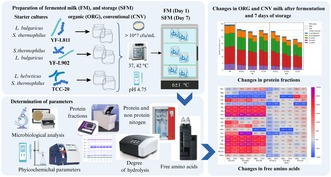

## 1 Introduction

Fermented milk (FM) is an important product category in the dairy sector because fermentation can improve shelf‐life stability, sensory properties, digestibility, and promote the formation of microbial metabolites derived from milk components (García‐Burgos et al. 2020; Marco et al. 2017). In yoghurt‐type products, fermentation is mainly driven by lactic acid bacteria (LAB), particularly *Streptococcus thermophilus* and *Lactobacillus delbrueckii* subsp. *bulgaricus*, which interact through protocooperation and contribute to the rapid acidification of the milk matrix (Leroy and De Vuyst 2004; Horiuchi and Sasaki 2012; Dan, Hu, Tai, et al. 2023). During fermentation, LAB metabolize lactose and nitrogen‐containing compounds, thereby influencing acidification, bacterial growth, gel formation, flavor development, and postfermentation changes during refrigerated storage (Liu et al. 2010; Kieliszek et al. 2021; Leroy and De Vuyst 2004).

Among these biochemical processes, proteolysis is particularly important because LAB require small peptides and free amino acids (FAAs) for growth in milk, which contains limited amounts of directly available low‐molecular‐weight nitrogen compounds (Savijoki et al. 2006; Liu et al. 2010; Kieliszek et al. 2021). The proteolytic system of LAB includes cell‐envelope proteinases, peptide transport systems, and intracellular peptidases, which together hydrolyse milk proteins into peptides and FAAs (Savijoki et al. 2006; Liu et al. 2010; Kieliszek et al. 2021). The intensity and specificity of proteolysis are species‐ and strain‐dependent because LAB differ in their enzymatic systems and in their ability to utilize casein‐derived peptides and amino acids (Savijoki et al. 2006; Liu et al. 2010). *Lactobacillus helveticus* is of particular interest in dairy fermentation because of its well‐developed proteolytic system and its capacity to release peptides with bioactive properties (Slattery et al. 2010; Griffiths and Tellez 2013). Therefore, starter culture (SC) composition is expected to be a major factor affecting protein fraction hydrolysis, proteolytic activity, and FAA formation in FM (Griffiths and Tellez 2013; Liu et al. 2010; Kieliszek et al. 2021).

In addition to SC composition, the milk matrix may influence fermentation by supplying lactose, proteins, minerals, fatty acids, peptides, amino acids, and other minor compounds that can affect microbial growth and enzyme activity (Schwendel et al. 2015; Sabunevica and Zagorska 2023). Organic (ORG) and conventional (CNV) milk are often comparable in major constituents such as total protein, fat, and lactose, but differences may occur in minor components, including whey protein fractions, vitamins, minerals, and fatty acid profiles (Brodziak et al. 2018; Manuelian et al. 2022; Schwendel et al. 2015). However, reported differences between ORG and CNV milk are not always consistent because milk composition is also influenced by breed, lactation stage, season, geographical location, herd management, and feeding system, including pasture access (Średnicka‐Tober et al. 2016; Schwendel et al. 2015; Manuelian et al. 2022). For this reason, ORG and CNV milk should be evaluated as different raw‐material models rather than as inherently superior or inferior substrates for fermentation (Schwendel et al. 2015; Manuelian et al. 2022; Sabunevica and Zagorska 2023).

ORG milk has frequently been associated with a more favorable lipid profile, including higher proportions of polyunsaturated fatty acids, n‐3 fatty acids, and conjugated linoleic acid, although these differences are strongly related to pasture grass intake and feeding regime (Butler et al. 2008; Florence et al. 2012; Średnicka‐Tober et al. 2016). ORG production systems also restrict the use of synthetic pesticides, growth‐promoting hormones, and routine prophylactic antibiotics, and some retail surveys have reported lower detection of certain production‐related residues in ORG milk than in CNV milk (Welsh et al. 2019). Nevertheless, this does not imply that CNV milk is unsuitable for fermentation since both ORG and CNV milk can serve as technologically appropriate substrates when they meet regulatory and dairy‐quality requirements (Schwendel et al. 2015; Welsh et al. 2019; Manuelian et al. 2022). Thus, the potential relevance of ORG milk in fermented dairy production should be considered mainly in relation to milk matrix composition, minor bioactive compounds, and possible interactions with SC metabolism rather than as a direct indication of superior fermented product quality (Schwendel et al. 2015; Sabunevica and Zagorska 2023).

To date, most comparative studies on ORG and CNV milk have focused on raw milk composition, fatty acid profile, nutritional quality, and production‐related factors (Schwendel et al. 2015; Średnicka‐Tober et al. 2016; Sabunevica and Zagorska 2023). Less attention has been paid to how ORG and CNV milk behave as substrates under controlled fermentation conditions using defined commercial SCs. Existing studies indicate that both milk types can be used for FM production, but the composition of the final product may depend on the interaction between milk matrix, SC, and refrigerated storage conditions (Florence et al. 2012; Sabunevica and Zagorska 2023). Therefore, distinguishing the effect of milk origin from the effects of SC composition and storage time is necessary for interpreting proteolysis‐related changes in FM (Liu et al. 2010; Schwendel et al. 2015; Kieliszek et al. 2021).

In the present study, multi‐supplier ORG and CNV milk collected over 1 year were used as two industrially relevant raw‐material models for FM production. Rather than assuming milk origin to be the dominant determinant of proteolysis, the study was based on the hypothesis that proteolysis‐related changes in FM would be more strongly associated with SC composition and refrigerated storage than with milk origin alone, while milk origin may modulate selected responses through interaction effects. Accordingly, the aim of this study was to evaluate the relative contributions of milk origin, commercial thermophilic SC composition, and 7 days of refrigerated storage to bacterial viability, proteolytic activity, protein fraction hydrolysis, and FAA profiles in FM.

## 2 Materials and Methods

### 2.1 Media, Chemicals and Reagents

MRS (Man, Rogosa, and Sharpe broth) and M17 broth were supplied by Scharlau (Barcelona, Spain), and cyclohexane solution was supplied by Biolife (Milan, Italia). Analytical grade chemicals and reagents: sodium hydroxide (NaOH), o‐phthalaldehyde (OPA), trichloroacetic acid (TCA), methanol (CH_3_OH), sodium tetraborate (B_4_Na_2_O_7_), β‐mercaptoethanol (β‐ME), Kjeldahl tablets, hydrogen peroxide (H_2_O_2_), sulfuric acid (H_2_SO_4_), hydrochloric acid (HCl), acetonitrile (MeCN), formic acid (HCOOH), 2‐propanol, proteins α‐casein (α‐CN), β‐casein (β‐CN), κ‐casein (κ‐CN), β‐lactoglobulin (β‐LG), α‐lactalbumin (α‐LA), bovine serum albumin (BSA), lactoferrin, and immunoglobulin G (IgG), denaturing solution (DS), and acetonitrile were obtained from Sigma–Aldrich (St. Louis, MO, USA). Protein 80 kit and reading chips were supplied by Agilent Technologies (Germany).

### 2.2 Milk, Starter Cultures and Preparation of FM

The commercial thermophilic SCs, consisting of *Lactobacillus delbrueckii* subsp. *bulgaricus*, *Streptococcus thermophilus* , or *Lactobacillus helveticus* , were obtained from Chr. Hansen (Denmark) (Table 1). Commercial bovine pasteurized milk (PM) of CNV and ORG origin was systematically purchased from a local dairy processing company (LTD Tukuma piens, Latvia) over 1 year. This company was selected because it processes both ORG and CNV milk sourced from numerous certified farms within the region, including seven ORG farms and 60 CNV farms. This enabled a multi‐supplier sampling approach representative of industrial practice. This sampling design allowed milk from both CNV and ORG agricultural systems to be compared under similar technological conditions, while minimizing the influence of factors such as region, delivery time, processing, heat treatment, and packaging. Samples were collected on four separate occasions, twice during the outdoor feeding period and twice during the indoor feeding period, in order to obtain a representative overview across the year. Seasonal variation was not considered as a separate experimental factor, since the aim of the study was to evaluate fermentation behavior and resulting hydrolysis products across the annual production period. On each sampling occasion, ORG and CNV milk samples were purchased on the same day, delivered to the laboratory, and fermented on the day of arrival. Four independent fermentations were carried out for each milk type.

**TABLE 1:** Starter cultures used in the study.

Sample abbreviation in the study	ORG/YFL811	ORG/YFL902	ORG/TCC20
	CNV/YFL811	CNV/YFL902	CNV/TCC20
Commercial name, Chr. Hansen	YF‐L811 YoFlex	YF‐L902 YoFlex	*TCC‐20*
Activity units	50 U	500 U	100 U
Physical state	Freeze‐dried	Frozen	Freeze‐dried
Recommended incubation temperature, °C	40–45	40–45	35–45
Incubation temperature applied, °C	41°C ± 1°C	41°C ± 1°C	37°C ± 1°C
Microorganisms	*Lactobacillus delbrueckii* subsp. *bulgaricus* *Streptococcus thermophilus*	*Streptococcus thermophilus Lactobacillus delbrueckii* subsp. *bulgaricus*	*Lactobacillus helveticus*, *Streptococcus thermophilus*
Starter characteristics	For very mild flavor, very high viscosity and very low postacidification	For very mild flavor, extra high viscosity and very low postacidification	For very mild flavor, medium postacidification

The use of milk from a single processor ensured comparable collection procedures, delivery time, heat treatment conditions, and packaging for both milk types. This design allowed the evaluation of proteolytic behavior under industrially relevant conditions while minimizing technological variability between the studied ORG and CNV milk samples.

Inoculation and fermentation were performed according to the starter producer's specifications, at the recommended inoculation rates, resulting in an initial inoculum of > 1 × 10^7^ mL^−1^ colony‐forming units (CFU), using pasteurized standardized milk after its arrival to the laboratory. The milk's proximate composition was analyzed using Milkoscan Mars (Foss Analytical, Hillerød, Denmark). ORG milk contained 2.05% ± 0.09% fat, 3.41% ± 0.19% protein, 4.75% ± 0.18% lactose, and CNV had 2.07% ± 0.06% fat, 3.34% ± 0.14% protein, 4.85% ± 0.16% lactose.

Fermentation was carried out in sterile, capped glass jars at 41°C ± 1°C (YFL811 and YFL902) and 37°C ± 1°C (TCC20) until the pH reached 4.75 ± 0.05. Samples were immediately cooled in an ice‐water bath and placed in the refrigerator at 6°C ± 1°C to mature for 12 h until reaching a final pH of 4.60 ± 0.05. Subsequently, FM samples were stored (6°C ± 1°C) for 7 days.

### 2.3 Analytical Methods

#### 2.3.1 pH Measurement and Lactic Acid Determination

The sample pH and titratable acidity were measured before, during, and after fermentation, maturation and during storage. The pH was measured with Seven Compact pH S220 pH‐meter (Mettler Toledo, USA) at room temperature (20°C ± 1°C) with standard calibration solutions (pH 4,7,9) (Funke Gerber, Germany). The titratable acidity of the FM samples was determined using 0.1 M NaOH solution. The concentration of lactic acid (%) was expressed using the equation as described by Li et al. (2022).

#### 2.3.2 Microbiological Analysis

The pour plate technique was used to determine CFU of LAB. For *L. helveticus* and *Lactobacillus delbrueckii* subsp. *bulgaricus*, MRS agar plates with 0.1% cycloheximide (4 μg mL^−1^) were used and were incubated anaerobically for 72 h at 37°C using GasPak EZ sachets (Becton, Dickinson and Company, USA). CFU of *S. thermophilus* were determined on M17 agar plates after incubation at 42°C for 48 h (IPP400, Memmert, Germany). Enumeration was performed by recording plates with 30–300 colonies using an automatic colony counter (Scan 500, Interscience International, France). Results are expressed as log_10_ CFU mL^−1^.

#### 2.3.3 Proteolytic Activity Measurement

The degree of protein hydrolysis was evaluated using the o‐phthaldialdehyde (OPA) method according to Church et al. (1983) with minor modifications. The OPA reagent was prepared in a prior analysis and maintained at 38°C ± 1°C in a water bath throughout the procedure to prevent solidification. For the assay, 2 g of each sample was mixed with 1 mL of deionized water and 5 mL of 5% TCA, incubated for 10 min, and centrifuged at 4.000 × g for 10 min at 4°C (PrO‐Research K2015R, Centurion Scientific, UK) to obtain the supernatant. Aliquots (100 μL) of supernatant were reacted with 2 mL of OPA reagent in the dark for 10 min at a temperature of 20°C ± 1°C. Absorbance was measured at 340 nm and 21°C ± 1°C using a UV–Vis spectrophotometer (UV‐1900i, Shimadzu Corporation, Japan). As a blank, 100 μL of deionized water and 2 mL of OPA were used.

#### 2.3.4 Total Protein and Nonprotein Nitrogen Determination

The content of total protein and nonprotein nitrogen compounds (NPNC) was determined by the Kjeldahl method using a nitrogen conversion factor of 6.38, according to ISO 8968‐1:^2014^. For NPNC determination, 50 mL of sample was heated at 95°C ± 1°C for 10 min, filtered through a 1 μm pore‐size filter (Whatman No. 1), and the resulting filtrate was collected and analyzed.

#### 2.3.5 Protein Fraction Analysis

##### 2.3.5.1 Samples and Standards

Prior to microfluidic chip electrophoresis analysis, PM and FM samples (1 g) were dissolved in 9 mL of deionized water. Protein standard solutions were prepared as described by Anema (2009). Briefly, individual protein standards were dissolved in deionized water (1 mg mL^−1^), and increased signals in the chip runs were used to identify kDa‐based peaks of each protein fraction (PF). Subsequently, a standard protein mixture was prepared by combining individual protein solutions, resulting in a final concentration of 1 mg mL^−1^ for each protein. This mixture and each individual protein standard solution were used to generate standard curves within the concentration range of 0.1–0.5 mg mL^−1^ (Figure S1). The equations with strong linearity (*R* ^2^ > 0.97) for whey proteins (β‐LG, α‐LA) and casein fractions (α‐, β‐, κ‐CN) were obtained. The replication of separation of the minor proteins (BSA, LF, IgG) was too low for accurate quantification; therefore, they were not applied.

##### 2.3.5.2 Analysis Using Microfluidic Chip Electrophoresis

Microfluidic chip electrophoresis was performed using an Agilent 2100 Bioanalyzer system with the associated Protein 80 kit and chips (Agilent Technologies, Germany). Gel matrix, destaining solution, denaturing solution, and samples (protein standards, PM, FM) were prepared according to the manufacturer's instructions (Agilent Technologies, Germany). For analysis, in 0.5 mL tubes, 4 μL of each sample was mixed with 2 μL of denaturing solution (Sigma‐Aldrich, USA), heated in a water bath (95°C ± 1°C, 5 min), immediately cooled on ice, briefly spun, and diluted with 84 μL of deionized water. Kit solutions (gel–dye matrix, destaining solution, molecular mass ladder) and samples were loaded onto the microfluidic chip, which was immediately inserted into the Bioanalyzer.

Quantification was based on the peak area in the electropherogram using the Agilent 2100 Expert software (Agilent Technologies, Germany).

#### 2.3.6 Free Amino Acid Determination

##### 2.3.6.1 Preparation of Free Amino Acids for HPLC‐ESI‐TQ‐MS/MS Analysis

FAAs were isolated and purified using a solid–liquid extraction procedure. In brief, 1.00 ± 0.01 g of sample was weighed into 15 mL centrifuge tubes, and 10.0 mL of 20% acidified acetonitrile (MeCN) (MeCN:H_2_O:HCOOH, 20:79:1, *v/v/v*) was added. The mixture was vortexed for 1 min, then sonicated (50 kHz, 300 W) for 30 min at 20°C ± 1°C using an “Ultrasons” ultrasonic bath (J.P. Selecta, Barcelona, Spain). After centrifugation (9280× *g*, 10 min, 4°C ± 1°C) in a Hermle Z 36 HK centrifuge (Hermle Labortechnik, GmbH, Wehingen, Germany), the supernatant was collected and filtered through a 0.22 polyvinylidene fluoride (PVDF) hydrophilic membrane filter (Durapore, Millipore, Billerica, MA, USA). For clean‐up and preconcentration, 1 mL of the filtrate was processed using an Amicon Ultra 2 mL centrifugal filter unit (3 kDa MWCO) by centrifugation at 3500 × *g* for 20 min at 19°C ± 1°C. The resulting filtrates were stored at −18°C ± 1°C and analyzed within 12 h.

##### 2.3.6.2 HPLC‐ESI‐TQ‐MS/MS Analysis of Free Amino Acids

FAAs were quantified by LC‐ESI‐MS/MS using the Shimadzu Nexera UC system coupled to a TQ‐MS‐8050 triple quadrupole, following the instrumentation settings and analytical conditions described in our previous work (Zagorska et al. 2024).

### 2.4 Data Analysis

Experiments were performed using four independent milk sampling occasions over 1 year. On each occasion, ORG and CNV PM were obtained on the same day and used for FM production with each SC. The FM sample produced from each milk origin × SC combination within each sampling occasion was considered the true experimental unit. Analytical measurements were performed in triplicate for each experimental unit, and the mean of the analytical triplicates was used for statistical analysis to avoid treating analytical replicates as independent biological replicates. A three‐way analysis of variance (ANOVA) was applied to assess the effects of milk origin (ORG, CNV), SC composition (YFL811, YFL902, TCC20), sampling time (PM, FM, SFM), and their interactions on the measured parameters. Prior to ANOVA, the assumptions of normality and homogeneity of variance were evaluated. When significant effects were detected, pairwise comparisons were performed using Tukey's post hoc test. Statistical significance was set at *p* < 0.05. Because the study was designed to evaluate overall proteolysis‐related responses across the annual sampling period rather than seasonal effects, sampling occasion was treated as an independent experimental repetition and was not included as a separate fixed factor. Data analysis and visualization were performed in Python 3.13.5.

## 3 Results

### 3.1 The Evaluation of Proteolysis Influencing Factors

Fermentation significantly affected acidification, bacterial counts, and proteolytic activity (at the individual level and in interactions with SC), with effects on most FAAs. During fermentation, selected SCs substantially contributed to changes in most of the studied parameters, considering bacterial counts, NPNC, proteolytic activity, and all FAAs, except for total protein and four PFs (α‐LA, β‐LG, β‐CN, and α‐CN) (Table S1). After 7 days of storage, the influence of the SC was no longer tangible for individual FAAs such as for Thr, Ala, Leu, and the sum of essential amino acids (EAAs), but it remained significant for changes in the other parameters (Table S2). Storage affected *Lactobacillus* spp., acidification, NPNC, Met, Glu, Tyr, and the sum of FAAs, the sum of PFs, and whey protein fractions. The interaction effect between applied study factors was also observed for *S. thermophilus* CFU, proteolytic activity, and most FAAs concentrations in SFM. On the whole, milk origin alone did not have a significant effect on the tested parameters during fermentation, but did affect bacterial counts and Met during storage. It should be noted that SC and sampling time, at both the individual and interaction levels, were related to most of the significant changes observed in this study and are discussed in detail below.

### 3.2 Control of the Fermentation Process and Changes in the Bacterial Counts

During fermentation, pronounced pH reductions were observed in YFL811 and TCC20 samples after the first hour, and the samples reached the final pH (4.75 ± 0.05) after 3.5–4.0 h. The YFL902 samples first showed a pH reduction after 2 h, and reached the final pH after 5 h of fermentation (Figure S2). After maturation, all samples reached pH 4.61 ± 0.02 with narrow variability across the SC (Table 2). After 7 days of storage, pH decreased steadily in all SFM samples, with no significant differences among them.

**TABLE 2:** Changes of pH in milk samples (*n* = 56).

Sample stage	YFL811		YFL902		TCC20	
	ORG	CNV	ORG	CNV	ORG	CNV
**pH**						
Inoculum	6.56 ± 0.14 Aa	6.59 ± 0.12 Aa	6.62 ± 0.12 Aa	6.64 ± 0.12 Aa	6.60 ± 0.11 Aa	6.62 ± 0.11 Aa
FM	4.60 ± 0.01 Ab	4.61 ± 0.01 ABb	4.62 ± 0.01 BCb	4.63 ± 0.01 ACb	4.62 ± 0.01 BCb	4.61 ± 0.01 ABCb
SFM	4.30 ± 0.09 Ac	4.29 ± 0.08 Ac	4.32 ± 0.02 Ac	4.39 ± 0.01 Ac	4.29 ± 0.03 Ac	4.32 ± 0.02 Ac
**Lactic acid, %**						
Inoculum	0.14 ± 0.01 Aa	0.15 ± 0.01 Aa	0.14 ± 0.00 Aa	0.13 ± 0.00 Aa	0.14 ± 0.00 Aa	0.14 ± 0.00 Aa
FM	0.77 ± 0.02 Ab	0.76 ± 0.02 Ab	0.74 ± 0.04 Ab	0.73 ± 0.08 Ab	0.74 ± 0.03 Ab	0.75 ± 0.04 Ab
SFM	0.90 ± 0.05 Ac	0.91 ± 0.01 Ac	0.92 ± 0.02 Ac	0.89 ± 0.00 Ab	0.88 ± 0.01 Ac	0.90 ± 0.01 Ac

*Note:* Data are expressed as mean ± standard deviation. Means with different uppercase letters (A–C) within the same row indicate significant differences among samples (milk origin × starter culture) (*p* < 0.05). Means with different lowercase letters (ac) within the same column indicate significant differences between sample stages (inoculum, FM, SFM) (*p* < 0.05) for the same sample.

Abbreviations: CNV, conventional; FM, fermented milk; ORG, organic; SFM, stored fermented milk (7 days); TCC20, TCC‐20; YFL811, YF‐L811 YoFlex; YFL902, YF‐L902 YoFlex.

During fermentation, LAB showed similar behavior in ORG and CNV samples, with a marked difference between the SCs (Table 3). In YFL902, the initial CFU immediately after inoculation were lower than those of YFL811 and TCC20. However, YFL902 demonstrated a pronounced increase in *S. thermophilus* CFU (Δlog_10_ 2.40 ± 0.04 CFU mL^−1^ in ORG/YFL902 and Δlog_10_ 2.36 ± 0.02 CFU mL^−1^ in CNV/YFL902, compared to Δlog_10_ 1.13–1.68 CFU mL^−1^ in TCC20 and YFL811), ultimately reaching the same *S. thermophilus* CFU as YFL811 in both ORG and CNV FM samples. In contrast, YFL902 exhibited lower growth of *Lactobacillus delbrueckii* subsp. *bulgaricus* in both milks (Δ log_10_ 0.46 ± 0.09 CFU mL^−1^ in ORG and Δlog_10_ 0.47 ± 0.19 CFU mL^−1^ in CNV samples), which was two‐fold lower compared to YFL811 (Δlog_10_ 0.96 ± 0.13 CFU mL^−1^ ORG/YFL811, Δlog_10_ 1.09 ± 0.08 CFU mL^−1^ CNV/YFL811) and three‐fold lower than in TCC20 (Δlog_10_ 1.20 ± 0.31 CFU mL^−1^ ORG/TCC20, Δlog_10_ 1.46 ± 0.05 CFU mL^−1^ CNV/TCC20). Notably, in the SFM samples, the stabilization of *Lactobacillus* spp. CFU in both ORG and CNV TCC20, but the reduction of *S. thermophilus* CFU only in CNV/TCC20 were observed. Among YFL811 and YFL902 SFM, the opposite behavior related to milk origin was established. The *S. thermophilus* CFU remained almost unchanged in ORG/YFL811 (Δ log_10_ 0.03 ± 0.01 CFU mL^−1^), whereas CNV/YFL811 samples showed a significant increase (Δ log_10_ 0.21 ± 0.01 CFU mL^−1^), resulting in significantly higher final CFU. In the ORG/YFL902, the *S. thermophilus* growth was more pronounced compared to the CNV/YFL902 FM samples (Δ 0.27 ± 0.05 vs.—Δ 0.05 ± 0.14 CFU mL^−1^), leading to higher CFU in ORG/YFL902 after the storage. In contrast, CNV/YFL902 samples exhibited slightly higher *Lactobacillus delbrueckii* subsp. *bulgaricus* growth, resulting in higher CFU compared to ORG/YFL902 SFM.

**TABLE 3:** Viable bacteria counts in fermented and stored fermented milk (*n* = 56).

Sample stage	YFL811		YFL902		TCC20	
	ORG	CNV	ORG	CNV	ORG	CNV
*Lactobacillus* spp., log_10_ CFU mL^−1^						
Inoculum	7.63 ± 0.04 ABa	7.70 ± 0.09 ABa	3.71 ± 0.10 Ca	3.58 ± 0.00 Ca	6.83 ± 0.45 ADa	6.96 ± 0.37 BDa
FM	8.60 ± 0.05 Ab	8.81 ± 0.02 Ab	4.16 ± 0.17 Bb	4.05 ± 0.00 Bab	8.11 ± 0.63 Ab	8.36 ± 0.47 Ab
SFM	8.73 ± 0.04 ABc	9.03 ± 0.03 Bc	4.51 ± 0.03 Cc	5.21 ± 0.59 Db	8.40 ± 0.01 ABb	8.32 ± 0.03 Ab
*Streptococcus thermophilus* , log_10_ CFU mL^−1^						
Inoculum	7.67 ± 0.19 Aa	7.79 ± 0.00 Aa	6.28 ± 0.37 Ba	6.58 ± 0.08 Ba	7.94 ± 0.00 Aa	7.52 ± 0.41 Aa
FM	8.95 ± 0.02 Ab	8.90 ± 0.01 Ab	8.89 ± 0.01 Ab	8.99 ± 0.01 Ab	9.30 ± 0.00 Bb	9.26 ± 0.07 Bb
SFM	8.98 ± 0.01 Ab	9.12 ± 0.02 BCDc	9.16 ± 0.04 Bb	8.94 ± 0.09 DEb	9.01 ± 0.06 CEc	9.19 ± 0.00 Bb

*Note:* Data are expressed as mean ± standard deviation. Means with different uppercase letters (A–C) within the same row indicate significant differences among samples (milk origin × starter culture) (*p* < 0.05). Means with different lowercase letters (a–c) within the same column indicate significant differences between sample stages (inoculum, FM, SFM) (*p* < 0.05) for the same sample.

Abbreviations: CNV, conventional; FM, fermented milk; ORG, organic; SFM, stored fermented milk (7 days); TCC20, TCC‐20; YFL811, YF‐L811 YoFlex; YFL902, YF‐L902 YoFlex.

### 3.3 Proteolysis

After fermentation, proteolytic activity increased by more than 2‐fold within all FM samples and continued to increase steadily during storage in all SFM (Table 4). The highest proteolytic activity was observed in SFM (CNV/TCC20), followed by CNV/YFL902 and ORG/YFL902. After 7 days of storage, samples fermented with YFL902 and TCC20 (specifically CNV/TCC20) showed stronger proteolytic activity than those fermented with YFL811.

**TABLE 4:** Proteolytic activity, total protein, and nonprotein nitrogen compound concentrations in pasteurized, fermented, and stored fermented milk (*n* = 56).

Sample stage	YFL811		YFL902		TCC20	
	ORG	CNV	ORG	CNV	ORG	CNV
Proteolytic activity, AU (340 nm)						
PM	68.50 ± 25.98 Aa	72.00 ± 25.64 Aa	68.50 ± 25.98 Aa	72.00 ± 25.64 Aa	68.50 ± 25.98 Aa	72.00 ± 25.64 Aa
FM	169.67 ± 24.52 Ab	158.83 ± 36.10 Ab	157.25 ± 4.57 Ab	167.00 ± 15.52 Ab	140.83 ± 15.42 Ab	155.00 ± 11.42 Ab
SFM	187.33 ± 21.46 Ab	191.50 ± 2.12 ABb	215.67 ± 2.31 ABCc	219.60 ± 14.31 BCc	199.40 ± 5.68 ABc	234.00 ± 14.27 Cc
NPNC, g 100 g^−1^						
PM	0.29 ± 0.02 Aa	0.29 ± 0.04 Aa	0.29 ± 0.02 Ab	0.29 ± 0.04 Aa	0.29 ± 0.02 Aa	0.29 ± 0.04 Aa
FM	0.34 ± 0.04 Aa	0.34 ± 0.03 Aa	0.34 ± 0.02 Aa	0.33 ± 0.00 Aa	0.32 ± 0.03 Aab	0.32 ± 0.03 Aa
SFM	0.33 ± 0.02 Aa	0.36 ± 0.06 ABa	0.35 ± 0.02 Aa	0.42 ± 0.03 Bb	0.35 ± 0.03 ABb	0.40 ± 0.03 ABb
Total protein, g 100 g^−1^						
PM	3.58 ± 0.32 Aa	3.56 ± 0.18 Aa	3.58 ± 0.32 Aa	3.56 ± 0.18 Aa	3.58 ± 0.32 Aa	3.56 ± 0.18 Aa
FM	3.78 ± 0.08 Aa	3.78 ± 0.17 Aa	3.72 ± 0.17 Aa	3.74 ± 0.21 Aa	3.73 ± 0.25 Aa	3.78 ± 0.13 Aa
SFM	3.77 ± 0.05 Aa	3.65 ± 0.10 Aa	3.64 ± 0.12 Aa	3.61 ± 0.08 Aa	3.66 ± 0.08 Aa	3.73 ± 0.08 Aa

*Note:* Data are expressed as mean ± standard deviation. Means with different uppercase letters (A–C) within the same row indicate significant differences among samples (milk origin × starter culture) (*p* < 0.05). Means with different lowercase letters (a–c) within the same column indicate significant differences between sample stages (PM, FM, SFM) (*p* < 0.05) for the same sample.

Abbreviations: AU, absorbance units; CNV, conventional; FM, fermented milk; NPNC, nonprotein nitrogen compounds; ORG, organic; PM, pasteurized milk; SFM, stored fermented milk (7 days); TCC20, TCC‐20; YFL811, YF‐L811 YoFlex; YFL902, YF‐L902 YoFlex.

### 3.4 Protein Fractions

**FIGURE 1:**
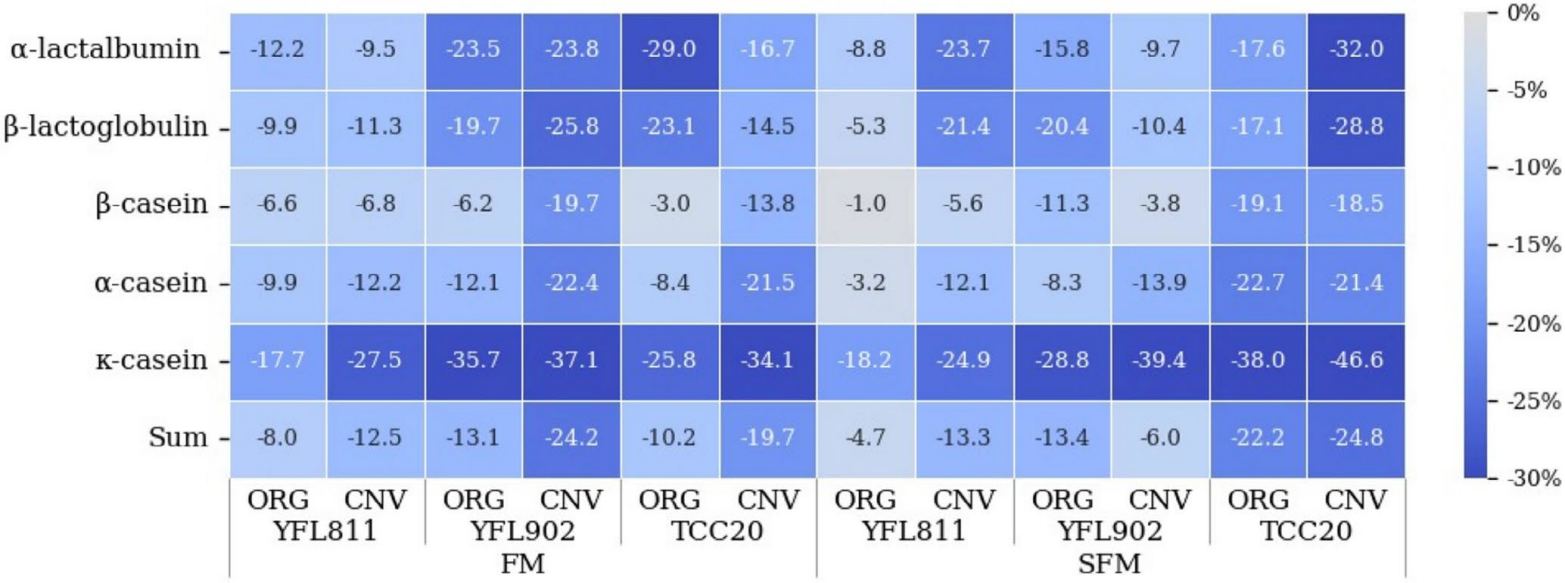
Heatmap of percentage changes (max decrease) in the identified protein fractions in organic (ORG) and conventional (CNV) fermented milk (FM) samples relative to pasteurized milk, and changes during storage (SFM) relative to FM.

The obtained PF concentrations (Table 5) showed no significant difference between ORG and CNV PM. The casein and whey PF content fell within the range reported by Amalfitano et al. (2020) and Wang et al. (2025). In FM samples, the relative ratio between casein fractions and whey proteins remained comparable to that in PM (Figure S3). After the fermentation, reduction between 8.0% and 24.2% of total PFs concentration (sum) across the samples was observed (Figure 1). Among applied SCs of the current study, YFL902 showed the most pronounced hydrolysis after fermentation, particularly in CNV/YFL902, with a total decrease of 24.2% in the concentrations of identified PFs, meanwhile the reduction in ORG/YFL902 was only 13.1%. Among identified PFs, the lowest level of degradation was observed in β‐CN and α‐CN, while the most pronounced decrease was observed in κ‐CN concentrations across all FM samples. After 7 days of storage, the decrease in κ‐CN concentration remained most pronounced among all PFs. Across the selected SCs, SFM with TCC20 showed that the total PF concentration decreased, with reductions of 22% in ORG and 24% in CNV compared to the beginning of storage.

**TABLE 5:** Protein fractions in pasteurized, fermented and stored fermented milk (*n* = 56).

Sample stage	YFL811		YFL902		TCC20	
	ORG	CNV	ORG	CNV	ORG	CNV
α‐LA, g kg^−1^						
PM	0.88 ± 0.45 Aa	0.81 ± 0.36 Aa	0.88 ± 0.45 Aa	0.81 ± 0.36 Aa	0.88 ± 0.45 Aa	0.81 ± 0.36 Aa
FM	0.77 ± 0.29 Aa	0.73 ± 0.27 Aa	0.67 ± 0.18 Aa	0.61 ± 0.31 Aa	0.62 ± 0.31 Aa	0.67 ± 0.16 Aab
SFM	0.70 ± 0.23 Aa	0.55 ± 0.19 Aa	0.56 ± 0.26 Aa	0.55 ± 0.31 Aa	0.52 ± 0.14 Aa	0.46 ± 0.15 Ab
β‐LG, g kg^−1^						
PM	5.78 ± 3.06 Aa	5.43 ± 2.86 Aa	5.78 ± 3.06 Aa	5.43 ± 2.86 Aa	5.78 ± 3.06 Aa	5.43 ± 2.86 Aa
FM	5.20 ± 2.52 Aa	4.82 ± 2.03 Aa	4.64 ± 1.68 Aa	4.02 ± 2.02 Aa	4.45 ± 1.89 Aa	4.64 ± 1.22 Aa
SFM	4.93 ± 1.51 Aa	3.78 ± 1.64 Aa	3.70 ± 2.03 Aa	3.61 ± 1.95 Aa	3.69 ± 1.23 Aa	3.31 ± 1.41 Aa
β‐CN, g kg^−1^						
PM	9.98 ± 3.79 Aa	9.89 ± 3.28 Aa	9.98 ± 3.79 Aa	9.89 ± 3.28 Aa	9.98 ± 3.79 Aa	9.89 ± 3.28 Aa
FM	9.32 ± 3.68 Aa	9.21 ± 3.25 Aa	9.36 ± 2.58 Aa	7.93 ± 3.57 Aa	9.68 ± 3.57 Aa	8.53 ± 2.45 Aa
SFM	9.22 ± 2.45 Aa	8.70 ± 2.55 Aa	8.31 ± 3.32 Aa	7.63 ± 3.12 Aa	7.83 ± 1.84 Aa	6.95 ± 1.99 Aa
α‐CN, g kg^−1^						
PM	12.39 ± 5.07 Aa	12.71 ± 5.22 Aa	12.39 ± 5.07 Aa	12.71 ± 5.22 Aa	12.39 ± 5.07 Aa	12.71 ± 5.22 Aa
FM	11.17 ± 4.38 Aa	11.16 ± 3.92 Aa	10.89 ± 2.93 Aa	9.87 ± 4.21 Aa	11.36 ± 4.21 Aa	9.98 ± 3.00 Aa
SFM	10.81 ± 3.04 Aa	9.81 ± 3.25 Aa	10.00 ± 4.29 Aa	8.50 ± 3.55 Aa	8.77 ± 2.43 Aa	7.85 ± 2.45 Aa
κ‐CN, g kg^−1^						
PM	4.26 ± 1.43 Aa	4.56 ± 1.25 Aa	4.26 ± 1.43 Aa	4.56 ± 1.25 Aa	4.26 ± 1.43 Aa	4.56 ± 1.25 Aa
FM	3.50 ± 1.23 Aab	3.31 ± 1.00 Aab	2.73 ± 0.89 Ab	2.87 ± 1.34 Ab	3.16 ± 0.94 Aab	3.01 ± 0.50 Ab
SFM	2.87 ± 0.69 Ab	2.49 ± 0.68 ABb	1.95 ± 0.65 Bb	1.74 ± 0.51 Bb	1.96 ± 0.81 Bb	1.61 ± 0.57 Bc
Sum of identified PFs, g kg^−1^						
PM	32.58 ± 12.17 Aa	33.40 ± 12.56 Aa	32.58 ± 12.17 Aa	33.40 ± 12.56 Aa	32.58 ± 12.17 Aa	33.40 ± 12.56 Aa
FM	29.97 ± 11.81 Aa	29.23 ± 10.02 Aa	28.31 ± 7.65 Aa	25.32 ± 10.98 Aa	29.26 ± 9.77 Aa	26.84 ± 6.87 Aab
SFM	28.56 ± 7.65 Aa	25.34 ± 7.35 Aa	24.51 ± 10.04 Aa	23.79 ± 7.49 Aa	22.76 ± 6.23 Aa	20.17 ± 6.12 Ab

*Note:* Data are expressed as mean ± standard deviation. Means with different uppercase letters (A–C) within the same row indicate significant differences among samples (milk origin × starter culture) (*p* < 0.05). Means with different lowercase letters (a–c) within the same column indicate significant differences between sample stages (PM, FM, and SFM) (*p* < 0.05) for the same sample.

Abbreviations: CNV, conventional; FM, fermented milk; ORG, organic; PM, pasteurized milk; SFM, stored fermented milk (7 days); TCC20, TCC‐20; YFL811, YF‐L811 YoFlex; YFL902, YF‐L902 YoFlex.

### 3.5 Free Amino Acids

After the fermentation, all FM samples exhibited a significant increase in total FAAs (sum of identified FAAs), branched‐chain amino acids (BCAAs), and EAAs (Table 6). Among individual FAAs, Ala, Leu, Phe, and Pro showed the most pronounced increases across all FM, whereas Glu, Gly, and Asp decreased after the fermentation. Across the SCs after the fermentation, a relatively (%) low increase of total BCAAs and EAAs was observed in TCC20 FM in both ORG and CNV samples, as well as in CNV/YFL811 FM samples. In turn, the relatively high increase of FAAs concentrations was observed in ORG/YFL811 samples, followed by YFL902/ORG and YFL902/CNV FM samples (Figure S4). The observed differences in concentrations might be attributed to the species and strain‐specific proteolytic activity of the LAB applied in the current study.

**TABLE 6:** Free amino acids in pasteurized, fermented and stored fermented milk (*n* = 56).

FAA, mg kg^−1^	YFL811		YFL902		TCC20	
	ORG	CNV	ORG	CNV	ORG	CNV
**Ser**						
PM	1.22 ± 0.41 Aa	1.22 ± 0.15 Aa	1.22 ± 0.41 Aa	1.22 ± 0.15 Aa	1.22 ± 0.41 Aa	1.22 ± 0.15 Aa
FM	1.07 ± 0.52 Aa	1.07 ± 0.37 Aa	1.67 ± 0.50 ABa	1.78 ± 0.35 ABa	1.94 ± 0.19 Bab	2.44 ± 0.18 Bb
SFM	0.52 ± 0.61 Aa	0.81 ± 0.65 Aa	1.78 ± 0.55 ABa	1.63 ± 0.34 ABa	2.80 ± 1.22 Bb	2.98 ± 0.82 Bb
**Asp**						
PM	2.99 ± 0.32 Aa	3.11 ± 0.49 Aa	2.99 ± 0.32 Aa	3.11 ± 0.49 Aa	2.99 ± 0.32 Aa	3.11 ± 0.49 Aa
FM	3.13 ± 1.64 Aa	2.58 ± 1.02 ABa	1.13 ± 0.45 Bb	1.18 ± 0.45 ABb	1.00 ± 0.37 Bb	1.05 ± 0.48 Bb
SFM	2.40 ± 0.89 ABa	2.61 ± 1.15 Ba	1.06 ± 0.45 ABb	1.03 ± 0.40 Ab	0.95 ± 0.42 Ab	1.00 ± 0.49 Ab
**Gly**						
PM	6.15 ± 1.04 Aa	6.07 ± 0.60 Aa	6.15 ± 1.04 Aa	6.07 ± 0.60 Aa	6.15 ± 1.04 Aa	6.07 ± 0.60 Aa
FM	0.86 ± 1.01 Ab	0.74 ± 0.89 Ab	0.41 ± 0.48 Ab	0.45 ± 0.52 Ab	1.02 ± 1.23 Ab	0.60 ± 0.69 Ab
SFM	0.94 ± 1.09 ABb	0.80 ± 0.94 ABb	0.00 ± 0.00 Bb	0.00 ± 0.00 Bb	3.63 ± 2.79 Aab	1.22 ± 1.41 ABb
**Thr**						
PM	1.80 ± 0.44 Aa	1.76 ± 0.33 Aa	1.80 ± 0.44 Aa	1.76 ± 0.33 Aa	1.80 ± 0.44 Aa	1.76 ± 0.33 Aa
FM	5.36 ± 2.50 Ab	4.27 ± 1.90 Aa	4.16 ± 0.63 Ab	4.64 ± 0.48 Ab	3.30 ± 0.31 Aab	2.88 ± 0.38 Aa
SFM	3.98 ± 1.67 Aab	4.01 ± 2.47 Aa	4.54 ± 1.28 Ab	4.95 ± 1.00 Ab	4.51 ± 1.93 Ab	4.80 ± 2.64 Aa
**Glu**						
PM	81.10 ± 9.43 Aa	81.15 ± 9.16 Aa	81.10 ± 9.43 Aa	81.15 ± 9.16 Aa	81.10 ± 9.43 Aa	81.15 ± 9.16 Aa
FM	57.65 ± 3.29 Ab	50.90 ± 2.00 ABb	51.84 ± 2.89 ABb	48.02 ± 1.68 ABb	40.86 ± 10.72 Bb	45.82 ± 9.58 ABb
SFM	4.88 ± 0.43 Ac	6.66 ± 1.66 Ac	54.69 ± 11.14 Bb	47.37 ± 1.49 Bb	4.36 ± 1.26 Ac	4.42 ± 1.00 Ac
**His**						
PM	0.63 ± 0.38 Aa	0.58 ± 0.37 Aa	0.63 ± 0.38 Aa	0.58 ± 0.37 Aa	0.63 ± 0.38 Aa	0.58 ± 0.37 Aa
FM	2.36 ± 1.21 Ab	2.10 ± 0.63 Ab	2.79 ± 0.47 Ab	2.93 ± 0.41 Ab	2.09 ± 0.42 Aab	1.90 ± 0.18 Aab
SFM	1.80 ± 0.65 Aab	1.91 ± 0.63 Ab	2.83 ± 0.60 Ab	2.85 ± 0.55 Ab	2.91 ± 1.48 Ab	2.98 ± 1.45 Ab
**Ala**						
PM	4.46 ± 0.82 Aa	4.53 ± 0.70 Aa	4.46 ± 0.82 Aa	4.53 ± 0.70 Aa	4.46 ± 0.82 Aa	4.53 ± 0.70 Aa
FM	17.59 ± 5.50 Ab	19.16 ± 8.45 Aab	18.32 ± 2.60 Ab	18.51 ± 2.01 Ab	17.02 ± 2.62 Ab	19.82 ± 5.72 Ab
SFM	19.68 ± 9.98 Ab	23.18 ± 12.25 Ab	20.56 ± 3.04 Ab	22.72 ± 0.25 A**c**	20.24 ± 0.52 Ac	23.55 ± 3.49 Ab
**Pro**						
PM	3.79 ± 0.66 Aa	4.17 ± 0.32 Aa	3.79 ± 0.66 Aa	4.17 ± 0.32 Aa	3.79 ± 0.66 Aa	4.17 ± 0.32 Aa
FM	29.27 ± 4.06 ABb	23.49 ± 1.16 Bb	34.14 ± 4.11 Ab	32.93 ± 2.10 Ab	31.48 ± 3.66 Ab	29.92 ± 3.94 ABb
SFM	23.99 ± 0.74 Ac	24.87 ± 4.25 Ab	34.10 ± 0.30 Bb	34.53 ± 1.31 Bb	36.41 ± 3.42 Bb	36.36 ± 4.72 Bb
**Arg**						
PM	3.74 ± 0.08 Aa	3.72 ± 0.12 Aa	3.74 ± 0.08 Aa	3.72 ± 0.12 Aa	3.74 ± 0.08 Aa	3.72 ± 0.12 Aa
FM	4.28 ± 1.37 Aa	3.10 ± 0.57 Aa	3.01 ± 0.48 Aa	3.20 ± 0.51 Aa	4.45 ± 1.04 Aa	3.95 ± 0.37 Aa
SFM	3.08 ± 0.17 Aa	3.23 ± 0.71 Aa	3.20 ± 0.48 Aa	3.27 ± 0.58 Aa	5.40 ± 2.12 Aa	5.86 ± 2.11 Aa
**Lys**						
PM	1.80 ± 0.18 Aa	1.79 ± 0.09 Aa	1.80 ± 0.18 Aa	1.79 ± 0.09 Aa	1.80 ± 0.18 Aa	1.79 ± 0.09 Aa
FM	6.03 ± 2.91 Ab	4.22 ± 1.32 ABb	2.14 ± 0.86 Ba	1.83 ± 0.26 Ba	3.14 ± 0.79 ABb	2.26 ± 0.41 Bab
SFM	3.98 ± 0.69 Aab	3.74 ± 1.01 Ab	1.90 ± 0.46 Ba	1.82 ± 0.22 Ba	2.98 ± 0.63 ABb	2.78 ± 0.74 ABb
**Val**						
PM	1.90 ± 0.20 Aa	1.89 ± 0.18 Aa	1.90 ± 0.20 Aa	1.89 ± 0.18 Aa	1.90 ± 0.20 Aa	1.89 ± 0.18 Aa
FM	4.25 ± 2.20 ABa	3.47 ± 1.27 ABa	4.13 ± 0.46 ABb	4.98 ± 0.25 Bb	2.48 ± 0.07 Aab	2.24 ± 0.54 Aa
SFM	2.96 ± 1.21 Aa	3.19 ± 1.68 Aa	4.08 ± 0.85 Ab	5.08 ± 0.78 Ab	2.67 ± 0.47 Ab	2.86 ± 1.46 Aa
**Met**						
PM	0.93 ± 0.01 Aa	0.95 ± 0.00 Aa	0.93 ± 0.01 Aa	0.95 ± 0.00 Aa	0.93 ± 0.01 Aa	0.95 ± 0.00 Aa
FM	1.30 ± 0.08 ABb	1.16 ± 0.02 ACb	0.91 ± 0.01 Da	1.00 ± 0.10 BCDa	0.00 ± 0.00 Eb	0.00 ± 0.00 Eb
SFM	0.97 ± 0.00 Aa	0.97 ± 0.01 Aa	0.00 ± 0.00 Bb	0.00 ± 0.00 Bb	0.96 ± 0.01 Aa	0.00 ± 0.00 Bb
**Tyr**						
PM	0.59 ± 0.22 Aab	0.56 ± 0.25 Aa	0.59 ± 0.22 Aa	0.56 ± 0.25 Aa	0.59 ± 0.22 Aa	0.56 ± 0.25 Aa
FM	1.35 ± 0.72 Aa	1.25 ± 0.02 Ab	6.44 ± 1.00 Bb	6.79 ± 0.66 Bb	0.70 ± 0.10 Aa	0.66 ± 0.07 Aa
SFM	0.25 ± 0.29 Ab	0.30 ± 0.34 Aa	4.30 ± 0.41 Bc	4.48 ± 1.51 Bc	0.57 ± 0.66 Aa	0.89 ± 1.02 Aa
**Leu**						
PM	0.84 ± 0.39 Aa	0.86 ± 0.33 Aa	0.84 ± 0.39 Aa	0.86 ± 0.33 Aa	0.84 ± 0.39 Aa	0.86 ± 0.33 Aa
FM	4.31 ± 2.16 Ab	3.06 ± 0.96 Ab	3.42 ± 0.37 Ab	3.92 ± 0.60 Ab	3.96 ± 0.13 Ab	3.49 ± 0.35 Ab
SFM	2.70 ± 0.69 Aab	3.07 ± 1.38 Ab	3.46 ± 0.64 Ab	4.22 ± 0.99 Ab	4.14 ± 0.73 Ab	4.67 ± 2.22 Ab
**Ile**						
PM	0.93 ± 0.40 Aa	0.96 ± 0.33 Aa	0.93 ± 0.40 Aa	0.96 ± 0.33 Aa	0.93 ± 0.40 Aa	0.96 ± 0.33 Aa
FM	5.33 ± 1.83 ABb	3.46 ± 1.52 ABa	4.14 ± 0.65 ABb	5.90 ± 1.59 Bb	3.34 ± 0.86 ABb	2.88 ± 0.32 Ab
SFMs	4.06 ± 0.88 Ab	3.68 ± 2.32 Aa	4.40 ± 0.07 Ab	7.76 ± 0.77 Bb	3.33 ± 0.25 Ab	3.17 ± 1.24 Ab
**Phe**						
PM	0.74 ± 0.40 Aa	0.74 ± 0.36 Aa	0.74 ± 0.40 Aa	0.74 ± 0.36 Aa	0.74 ± 0.40 Aa	0.74 ± 0.36 Aa
FM	4.13 ± 1.42 ABb	3.44 ± 0.88 Ab	5.32 ± 0.14 Bb	5.37 ± 0.58 Bb	3.92 ± 0.14 ABb	3.74 ± 0.47 ABb
SFM	2.76 ± 0.79 Ab	3.04 ± 1.37 Ab	5.49 ± 0.25 BCb	5.82 ± 1.15 Bb	3.39 ± 0.14 Ac	3.71 ± 0.84 ACb
**EAA**						
PM	9.10 ± 2.85 Aa	9.04 ± 2.47 Aa	9.10 ± 2.85 Aa	9.04 ± 2.47 Aa	9.10 ± 2.85 Aa	9.04 ± 2.47 Aa
FM	32.40 ± 14.96 Ab	24.61 ± 9.15 Aa	26.56 ± 2.78 Ab	30.08 ± 1.55 Ab	22.24 ± 1.01 Ab	19.38 ± 2.60 Aab
SFM	22.71 ± 7.11	23.14 ± 11.40	26.69 ± 4.12	32.50 ± 5.40	24.40 ± 5.66	24.97 ± 10.56
**BCAA**						
PM	3.67 ± 0.98 Aa	3.70 ± 0.83 Aa	3.67 ± 0.98 Aa	3.70 ± 0.83 Aa	3.67 ± 0.98 Aa	3.70 ± 0.83 Aa
FM	13.88 ± 6.18 Ab	10.00 ± 3.75 Ab	11.69 ± 0.38 Ab	14.80 ± 0.77 Ab	9.78 ± 0.78 Ab	8.60 ± 1.20 Ab
SFM	9.71 ± 2.77 Ab	9.94 ± 5.38 Ab	11.94 ± 1.55 Ab	17.05 ± 2.53 Ab	10.14 ± 0.95 Ab	10.70 ± 4.92 Ab
**Sum**						
PM	113.16 ± 3.69 Aa	113.58 ± 5.21 Aa	113.16 ± 3.69 Aa	113.58 ± 5.21 Aa	113.16 ± 3.69 Aa	113.58 ± 5.21 Aa
FM	147.58 ± 21.53 Ab	126.90 ± 6.48 Ab	143.52 ± 2.57 Ab	142.95 ± 3.04 Ab	120.71 ± 13.31 Aa	123.64 ± 15.25 Aa
SFM	78.46 ± 1.09 Ac	86.04 ± 6.05 Ac	146.38 ± 14.07 Bb	147.55 ± 10.84 Bb	98.77 ± 16.95 Aa	101.26 ± 15.85 Aa

*Note:* Data are expressed as mean ± SD. Means with different uppercase letters (A–C) within the same row indicate significant differences among samples (milk origin × starter culture) (*p* < 0.05). Means with different lowercase letters (a–c) within the same column indicate significant differences between sampling times (PM, FM, and SFM) (*p* < 0.05) for the same sample.

Abbreviations: BCAAs, total concentration of branched‐chain amino acids; CNV, conventional; EAAs, total concentration of essential amino acids; FM, fermented milk; ORG, organic; PM, pasteurized milk; SFM, stored fermented milk (7 days); Sum, total concentration of all identified FAAs; TCC20, TCC‐20; YFL811, YF‐L811 YoFlex; YFL902, YF‐L902 YoFlex.

**FIGURE 2:**
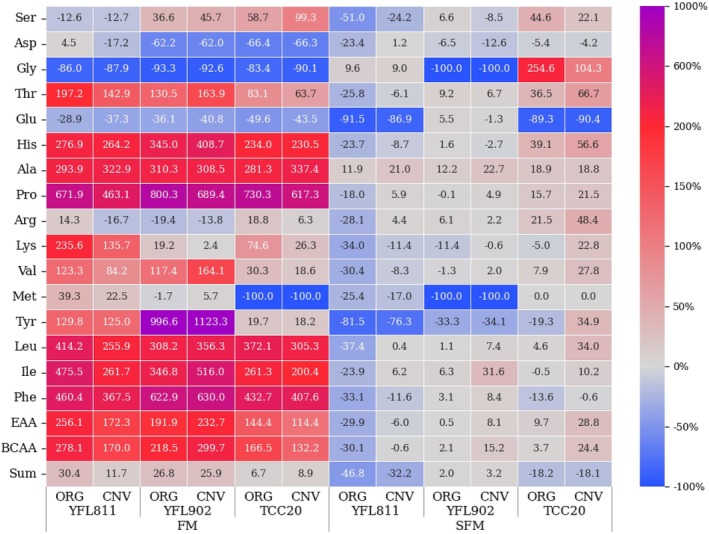
Heatmap of percentage (%) changes (max increase and max decrease) in free amino acids (FAAs) in organic (ORG) and conventional (CNV) fermented milk (FM) samples relative to pasteurized milk, and stored fermented milk (SFM) relative to FM. BCAA, total concentration of branched‐chain amino acids; CNV, conventional; EAA, total concentration of essential amino acids; ORG, organic; Sum, total concentration of all identified FAAs; TCC20, TCC‐20; YFL811, YF‐L811 YoFlex; YFL902, YF‐L902 YoFlex.

When comparing FAAs change (%) (Figure 2) based on milk origin, ORG/TCC20 had a more pronounced increase in EAAs, BCAAs, Phe, Lys, Arg, Thr, and Glu, whereas Gly and Ser increased the most in CNV/TCC20. The difference between ORG and CNV samples was more evident in YFL811 FM samples; ORG/YFL811 demonstrated a relatively higher concentration of all individual FAAs, except Glu, Gly, and Thr. The opposite tendency was observed in YFL902 FM samples: relatively higher Pro and Lys concentrations were detected in ORG FM samples, whereas BCAAs, Ile, Tyr, Leu, and Val were higher in CNV samples.

In SFM, a decrease in total FAAs concentration was observed across all samples, except those produced with YFL902. Such results could be explained by a significant decrease in Glu, the most abundant AA, with decreases of at least ten‐fold in TCC20 and YFL811 samples. The YFL902 samples with lower *Lactobacillus delbrueckii* subsp. *bulgaricus* bacterial counts showed a less pronounced reduction in Glu concentration than the YFL811 and TCC20 SFM samples.

## 4 Discussion

Changes in pH and lactic acid concentration are key indicators for monitoring the fermentation process and evaluating postacidification in SFM, and the results obtained in this study are consistent with those reported by Li et al. (2020). The obtained differences in acidification and LAB growth during the fermentation might be attributed to the composition and physical state of SC, and the observed growth pattern suggests a relatively stronger proliferation of *S. thermophilus* in YFL902 than in YFL811. Furthermore, frozen SC has a longer lag phase, leading to a delay in pH drop, as the *Lactobacillus* spp. generally exhibit a higher acidification rate in bovine milk compared to *S. thermophilus* (Warakaulle et al. 2025). Furthermore, ratios of 1:1 and 1:2 between *Lactobacillus delbrueckii* subsp. *bulgaricus* and *S. thermophilus* are typical for yoghurt SC, allowing these two bacteria to form a symbiotic relationship known as protocooperation (Dan, Hu, Tian, et al. 2023). During fermentation, *S. thermophilus* possesses higher metabolic activity and produces lactic acid, which stimulates the growth of *Lactobacillus delbrueckii* subsp. *bulgaricus*. Meanwhile, *Lactobacillus delbrueckii* subsp. *bulgaricus* releases peptides and FAAs from milk proteins, which are necessary for the development of *S. thermophilus* . This mutual exchange of nutrients improves fermentation efficiency and results in favorable texture, taste, and bacterial growth in yoghurt (Karagöl et al. 2026; Liu et al. 2016). *L. helveticus* positively affects *S. thermophilus* growth by shortening its lag phase, thereby reinforcing the observed high growth of *S. thermophilus* in TCC20. The interaction between these two bacteria is interdependent, as *S. thermophilus* supplies *L. helveticus* with CO_2_ and/or formic acid, which are necessary for its growth (Charlet et al. 2009). The observed differences between YFL902 and YFL811 in ORG and CNV samples suggest that interactions between *Lactobacillus* spp. and *S. thermophilus* may depend not only on starter culture composition but also on substrate‐related factors during FM storage. In this context, ORG and CNV milk appeared to modulate LAB growth in a starter‐dependent manner. Charlet et al. (2009) reported that undissociated lactic acid may inhibit LAB growth, whereas FAAs and/or small peptides in the substrate may have a growth‐stimulatory effect. Therefore, differences in the availability of nitrogenous compounds in milk and FM samples may partly explain the observed variation in LAB counts.

As previously reported by several authors, proteolytic activity increased during fermentation, and continued to increase steadily during refrigerated storage (Li et al. 2020; Karagöl et al. 2026). Among the LAB, *Lactobacillus* spp. possesses a higher proteolytic activity than *S. thermophilus* (Rajagopal and Sandine 1990). *Lactobacilli* possess the extracellular protease PrtB to utilize milk proteins, thereby liberating low‐molecular‐weight peptides and amino acids, which support the growth of *S. thermophilus* (Kunji et al. 1996). Therefore, more pronounced detected proteolytic activity in YFL902 FM samples could also be related to *S. thermophilus* ability to synthesize amino acids *de novo* (Liu et al. 2016). Compared to other *Lactobacillus* spp., which typically contain only one cell‐envelope proteinase, the presence of several cell‐envelope proteinases in *L. helveticus* makes it one of the most proteolytic LAB and the most effective in synthesizing a variety of bioactive peptides (Raveschot et al. 2020; Ter et al. 2024). Chelladhurai et al. (2025) showed that *L. helveticus* exhibits higher proteolytic activity due to its greater requirement for all amino acids than *Lactobacillus delbrueckii* subsp. *bulgaricus* and *S. thermophilus* . This coincides with our results only after 7 days of storage in СNV, but not in ORG FM TCC20 samples.

The similar results in NPNC changes after fermentation were obtained by Alm (1982), and the obtained results suggested qualitative changes in the composition of nitrogen‐containing compounds in the current study (Kieliszek et al. 2021). Evaluating PFs, Bonczar et al. (2016) also observed an 8%–19% reduction in the casein fractions, but only a 0.3%–2.0% reduction in α‐LA and β‐LG in yoghurt. The decrease in α‐LA after fermentation in YF902, consistent with Pescuma et al. (2008), who reported a similar reduction in α‐LA in yoghurt. The authors noted that α‐LA was more susceptible to hydrolysis than β‐LG, a finding also supported by the obtained results across all SCs. According to Liu et al. (2012), *Lactobacillus delbrueckii* subsp. *bulgaricus* 2038 first uses whey proteins as a nitrogen source rather than caseins. However, previous studies have revealed relatively low activity of *Lactobacillus delbrueckii* subsp. *bulgaricus* against bovine whey proteins, suggesting that strains used for yoghurt production can hydrolyse whey proteins only to a limited extent (Chandan et al. 1982; Bertrand‐Harb et al. 2003).

The decrease in κ‐CN is consistent with previously reported Bonczar et al. (2016) results, who observed the most pronounced changes after the FM storage. Nevertheless, Shi et al. (2014) reported reductions in the concentrations of four PFs (α‐CN, β‐CN, β‐LG, α‐LA) after 5 days of storage in FM with *Lactobacillus casei* N1115. The proteolytic system of LAB comprises three major components. The first is the cell wall‐bound proteinase that initiates the degradation of extracellular caseins in milk into oligopeptides (Liu et al. 2010). The proteinase activity varies among strains and species (Kieliszek et al. 2021) and further depends on the configuration of casein micelles in milk (Chandan et al. 1982). Bonczar et al. (2016) showed that *Lactobacillus* spp. have lower hydrolytic abilities against α‐CN compared to β‐CN, with *Lactobacillus acidophilus* reported to hydrolyse 26% β‐CN but only 14% α‐CN during fermentation. This is consistent with the results obtained in the current study. According to Chandan et al. (1982), *L. helveticus* and *Lactobacillus delbrueckii* subsp. *bulgaricus* both hydrolyse κ‐CN quicker than β‐CN, despite the fact that β‐CN was previously believed to be the most susceptible fraction in FM.

The elevated levels of Ala, Ile, Leu, Phe, Pro, and Thr observed in this study further support earlier findings of Warakaulle et al. (2025). The overall increase in FAAs could be explained by LAB proteolytic release and amino acid *de novo* synthesis to support their energy requirements, growth, and cell viability (Chelladhurai et al. 2025). Gu et al. (2021) highlighted Arg, Cys, Glu, Leu, Pro, and Thr as the most essential for the maintenance and growth of LAB. The presence of *Lactobacillus delbrueckii* subsp. *bulgaricus* in YFL811 and YFL902 stimulates *S. thermophilus* growth by releasing Val, His, Met, Glu, and Leu in YFL811 and YFL902 FM samples (Abu‐Tarboush 1996; Pescuma et al. 2008; Kieliszek et al. 2021). Although *L. helveticus* is known for its high proteolytic activity, the lower FAAs in TCC20 compared to other applied SC might be due to *L. helveticus's* exceptional requirements and utilization for all amino acids (Chelladhurai et al. 2025). Previously, Gu et al. (2021) reported that Glu is one of the most extensively metabolized AAs during fermentation, with its concentration reduced by 0.8–6.1‐fold, depending on the LAB species and strains used. Moreover, *Lactobacillus* spp. possesses higher glutamate decarboxylase activity, which allows them to convert Glu to γ‐aminobutyric acid (GABA) (Gu et al. 2021). The pronounced decrease in Glu observed in the present study is consistent with extensive Glu metabolism by LAB and may be linked to glutamate decarboxylase activity; nevertheless, since GABA was not measured, this interpretation remains tentative.

Although all species‐specific nutritional requirements of *Lactobacillus delbrueckii* subsp. *bulgaricus* are not fully defined, it is well established that His is supplied from oligopeptides, and Lys, Asp, Glu, and Ala are essential for its growth (Liu et al. 2016). The growth requirements of *S. thermophilus* include Ser, Leu, Ile, Val, Lys, and Arg, and the reduction of these FAAs during fermentation (Letort et al. 2002). However, *S. thermophilus* can grow in a substrate limited in BCAAs, as it can synthesize them via its endogenous metabolic pathways (Hervé‐Jiménez et al. 2009). This is consistent with the present results, where in YFL902 FM samples, *S. thermophilus* exhibited the most intense growth, and BCAAs concentrations were exhibited during storage. The higher concentration of aromatic amino acids (Phe, Tyr, Trp, Glu) (Kasperek et al. 2024) was detected in samples YFL811 and YFL902; starters could be recommended for obtaining FM with more pronounced flavor.

The FAA profiles showed that proteolysis in FM was strongly affected by the starter culture used. Samples fermented with YFL811 and YFL902 generally contained higher amounts of total FAAs, EAAs, and BCAAs than those fermented with TCC20, indicating differences in the extent of protein hydrolysis and amino acid release among the starter cultures. YFL902 showed a particularly pronounced hydrolysis pattern, whereas the effect of milk origin during fermentation was limited. Differences between ORG and CNV samples became more apparent after refrigerated storage, suggesting that milk origin may influence selected storage‐related changes. For example, the higher FAA retention observed in ORG/YFL902 SFM may be related to interactions between the milk matrix and LAB proteolytic activity. However, as digestibility, sensory properties, and bioactivity were not assessed in this study, these findings should be interpreted as changes in proteolysis‐related biochemical parameters rather than as evidence of improved nutritional or functional properties.

## 5 Conclusions

Fermentation increased proteolysis in all FM samples, resulting in the hydrolysis of protein fractions and the accumulation of free amino acids. Proteolysis continued during maturation and 7‐day refrigerated storage, as reflected by increased o‐phthaldialdehyde values and higher concentrations of nonprotein nitrogen compounds. Commercial starter culture composition and storage time were the main factors contributing to proteolysis‐related changes, whereas milk origin had a less pronounced effect during fermentation and mainly modulated selected responses after storage. Overall, organic and conventional milk behaved as comparable substrates for fermentation under the applied industrially relevant conditions. These findings indicate that starter‐dependent effects should be considered the dominant drivers of protein hydrolysis and nonprotein nitrogen metabolite formation in FM, while milk production system may contribute to selected matrix‐dependent differences. The results provide broader insight into proteolysis‐related changes in FM and may support the development of fermented dairy products through appropriate selection of starter cultures and consideration of milk raw‐material characteristics.

## Author Contributions

**Jelena Zagorska:** conceptualization, writing – original draft, project administration, supervision. **Kristine Majore:** writing – review and editing. **Stefanija Steinweg:** conceptualization, investigation, writing – original draft, formal analysis, data curation. **Inga Ciprovica:** validation, writing – review and editing. **Vitalijs Komasilovs:** data curation, visualization. **Vitalijs Radenkovs:** investigation, formal analysis, writing – review and editing.

## Funding

This research was supported by project No Nr. 5.2.1.1.i.0/2/24/I/CFLA/002 “Strengthening the Institutional Capacity of LBTU for Excellence in Studies and Research”, funded by The Recovery and Resilience Facility.

## Ethics Statement

The authors declare that the present manuscript does not involve any animal and human study.

## Conflicts of Interest

The authors declare no conflicts of interest.

## Supporting information


**Figure S1:** Separation of milk proteins by microfluidic chip electrophoresis. (a) Gel image, standard protein mixture (five proteins), at concentrations ranging 0.1–0.5 mg mL^−1^ [1–5]; (b) Gel image, fermented milk samples [1–8]; (c) Electropherogram of standard protein mixture (five proteins), at 1 mg mL^−1^; (d) electropherogram of fermented milk sample (ORG/YFL811).

**Figure S2:** pH changes in FM samples.

**Figure S3:** Protein fraction profile in organic (ORG) and conventional (CNV) pasteurized (PM), fermented (FM) and stored fermented milk (SFM). CNV, conventional; ORG, organic; TCC20, TCC‐20; YFL811, YF‐L811 YoFlex; YFL902, YF‐L902 YoFlex.

**Figure S4:** Free amino acid (FAA) profile in organic (ORG) and conventional (CNV) pasteurized milk (PM), fermented (FM) and stored fermented milk (SFM). CNV, conventional; FM, fermented milk; ORG, organic; SFM, stored fermented milk (7 days); TCC20, TCC‐20; YFL811, YF‐L811 YoFlex; YFL902, YF‐L902 YoFlex.

**Table S1:** Effect of applied factors on the study parameters during the fermentation.

**Table S2:** Effect of applied factors on the study parameters during the storage.

## Data Availability

The data supporting the findings of the current research are available from the corresponding author (Stefanija Steinweg) upon reasonable request.
