# Supplementary material for: Proteolytic Activity of Commercial Thermophilic Starter Cultures and Changes in Protein Fractions and Free Amino Acids in Organic and Conventional Fermented Milk
Source: Food Sci Nutr. 2026 Aug 2;14(8):e72199. doi: 10.1002/fsn3.72199 (PMC13429941; doi:10.1002/fsn3.72199)
Supplement: Supplementary file 1 — Figure S1: Separation of milk proteins by microfluidic chip electrophoresis. (a) Gel image, standard protein mixture (five proteins), at concentrations ranging 0.1–0.5 mg mL−1 [1–5]; (b) Gel image, fermented milk samples [1–8]; (c) Electropherogram of standard protein mixture (five proteins), at 1 mg mL−1; (d) electropherogram of fermented milk sample (ORG/YFL811). [file FSN3-14-e72199-s003.docx]

**
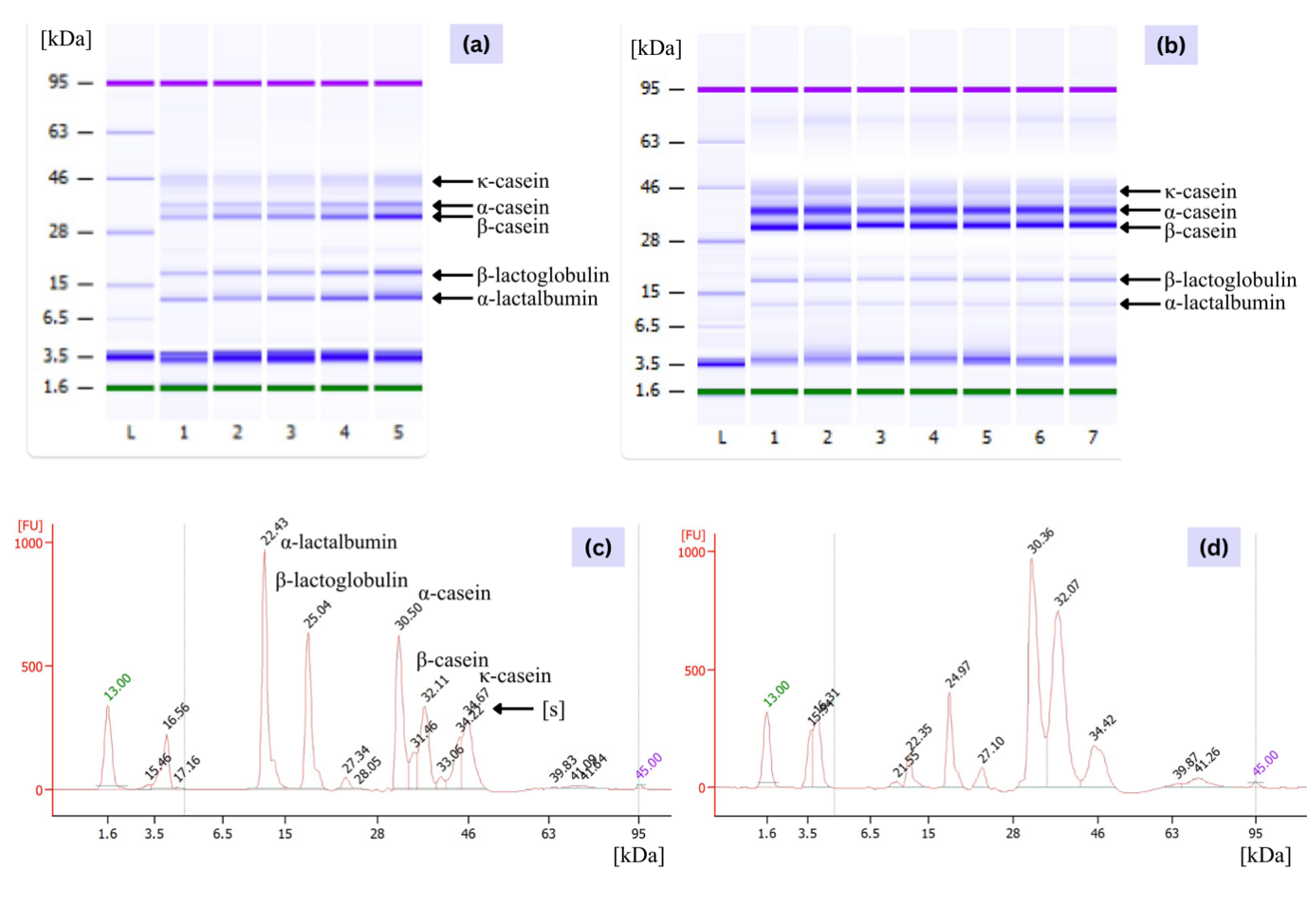
**

Supplementary Appendix **Figure 1** Separation of milk proteins by microfluidic chip electrophoresis. (**a) –** gel image, standard protein mixture (five proteins), at concentrations ranging 0.1–0.5 mg mL ^-1^ [**1-5**]; (**b) –** gel image, fermented milk samples [**1-8**]; (**c)** – electropherogram of standard protein mixture (five proteins), at 1 mg mL ^-1^_; (_**d) –**electropherogram of fermented milk sample (ORG/YFL811)
