# Supplementary figures and images for: Proteolytic Activity of Commercial Thermophilic Starter Cultures and Changes in Protein Fractions and Free Amino Acids in Organic and Conventional Fermented Milk
Source: Food Sci Nutr. 2026 Aug 2;14(8):e72199. doi: 10.1002/fsn3.72199 (PMC13429941; doi:10.1002/fsn3.72199)

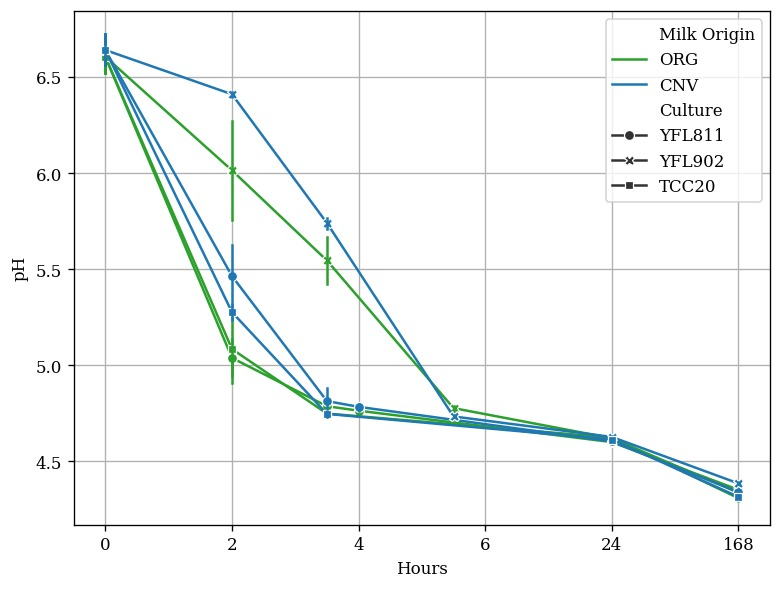


Supplementary Appendix **Figure 2** pH changes in FM samples

Supplement: Supplementary file 2 — Figure S2: pH changes in FM samples. [file FSN3-14-e72199-s001.docx]
