# Supplementary material for: Proteolytic Activity of Commercial Thermophilic Starter Cultures and Changes in Protein Fractions and Free Amino Acids in Organic and Conventional Fermented Milk
Source: Food Sci Nutr. 2026 Aug 2;14(8):e72199. doi: 10.1002/fsn3.72199 (PMC13429941; doi:10.1002/fsn3.72199)
Supplement: Supplementary file 3 — Figure S3: Protein fraction profile in organic (ORG) and conventional (CNV) pasteurized (PM), fermented (FM) and stored fermented milk (SFM). CNV, conventional; ORG, organic; TCC20, TCC‐20; YFL811, YF‐L811 YoFlex; YFL902, YF‐L902 YoFlex. [file FSN3-14-e72199-s005.docx]

**
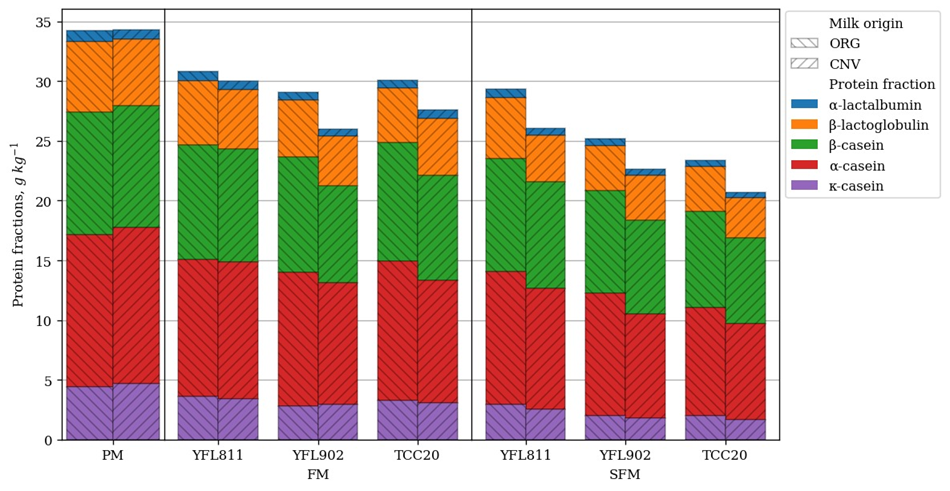
**

Supplementary Appendix **Figure 3** Protein fraction profile in organic (ORG) and conventional (CNV) pasteurised (PM), fermented (FM) and stored fermented milk (SFM)

Abbreviations: YFL811 – YF-L811 YoFlex, YFL902 –YF-L902 YoFlex, TCC20 – TCC-20; ORG – organic, CNV – conventional
