# Supplementary material for: Proteolytic Activity of Commercial Thermophilic Starter Cultures and Changes in Protein Fractions and Free Amino Acids in Organic and Conventional Fermented Milk
Source: Food Sci Nutr. 2026 Aug 2;14(8):e72199. doi: 10.1002/fsn3.72199 (PMC13429941; doi:10.1002/fsn3.72199)
Supplement: Supplementary file 4 — Figure S4: Free amino acid (FAA) profile in organic (ORG) and conventional (CNV) pasteurized milk (PM), fermented (FM) and stored fermented milk (SFM). CNV, conventional; FM, fermented milk; ORG, organic; SFM, stored fermented milk (7 days); TCC20, TCC‐20; YFL811, YF‐L811 YoFlex; YFL902, YF‐L902 YoFlex. [file FSN3-14-e72199-s004.docx]

**
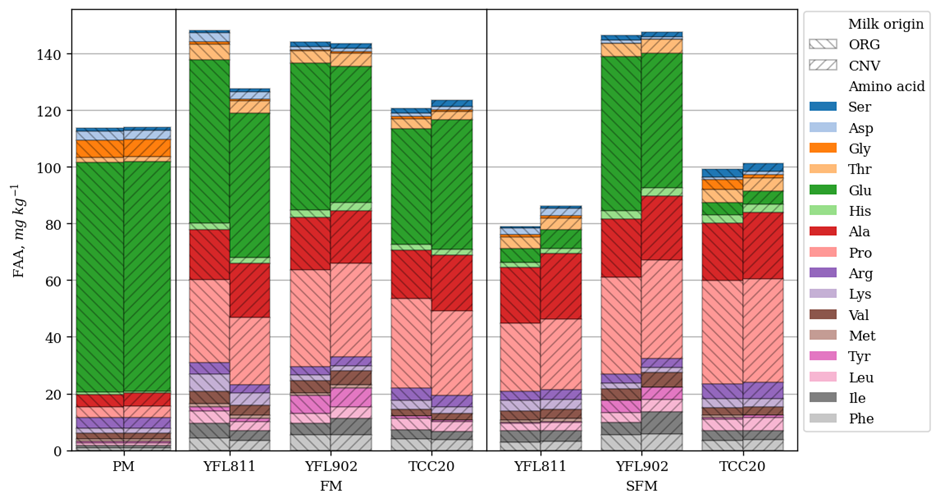
**

Supplementary Appendix **Figure 4** Free amino acid (FAA) profile in organic (ORG) and conventional (CNV) pasteurised milk (PM), fermented (FM) and stored fermented milk (SFM)

Abbreviations: FM – fermented milk, SFM – stored fermented milk (7 days), YFL811 – YF-L811 YoFlex, YFL902 –YF-L902 YoFlex, TCC20 – TCC-20; ORG – organic, CNV – conventional
