# Supplementary material for: Proteolytic Activity of Commercial Thermophilic Starter Cultures and Changes in Protein Fractions and Free Amino Acids in Organic and Conventional Fermented Milk
Source: Food Sci Nutr. 2026 Aug 2;14(8):e72199. doi: 10.1002/fsn3.72199 (PMC13429941; doi:10.1002/fsn3.72199)
Supplement: Supplementary file 5 — Table S1: Effect of applied factors on the study parameters during the fermentation. [file FSN3-14-e72199-s002.docx]

Supplementary Appendix **Table 1** Effect of applied factors on the study parameters during the fermentation

| **Parameter** | Study factors and their interactions | | | | | | |
| --- | --- | --- | --- | --- | --- | --- | --- |
|  | **SC** | **MO** | **T** | **MO**×**SC** | **SC**×**T** | **MO**×**T** | **MO**×**SC**×**T** |
| CFU, *Lactobacillus spp.* | 0.000 | NS | 0.000 | NS | 0.019 | NS | NS |
| CFU, *S. thermophilus* | 0.000 | NS | 0.000 | NS | 0.000 | NS | NS |
| Lactic acid | NS | NS | 0.000 | NS | NS | NS | NS |
| pH | NS | NS | 0.000 | NS | NS | NS | NS |
| Total protein | NS | NS | NS | NS | NS | NS | NS |
| NPNC | 0.008 | NS | NS | NS | NS | NS | NS |
| Proteolytic activity | 0.000 | NS | 0.023 | NS | NS | NS | NS |
| FAAs – Asp, Gly, Thr, Glu, Glu, His, Lys, Val, Leu, Phe, Sum of BCAAs, Sum of EAAs, Sum of FAAs | 0.000 | NS | NS | NS | 0.009-0.048 | NS | NS |
| FAAs – Ala, Arg, Met | 0.000 | NS | NS | NS | NS | NS | NS |
| FAAs – Pro, Tyr | 0.000 | NS | NS | NS | NS | 0.011, 0.014 | NS |
| FAAs – Ile | 0.000 | NS | NS | 0.025 | 0.016 | NS | NS |
| Sum of PFs | NS | NS | NS | NS | NS | NS | NS |
| PFs – α-LA, β-LG, β-CN, α-CN | NS | NS | NS | NS | NS | 0.002-0.041 | NS |
| PF - κ-CN | 0.008 | NS | NS | NS | NS | NS | NS |
| Abbreviations: MO – milk origin; SC – starter culture; T – sampling time; CFU – colony founding units; NPNC – non-protein nitrogen compounds; FAAs – free amino acids; PFs – protein fractions; NS – not significant (p>0.05). | | | | | | | |
