# Supplementary material for: Proteolytic Activity of Commercial Thermophilic Starter Cultures and Changes in Protein Fractions and Free Amino Acids in Organic and Conventional Fermented Milk
Source: Food Sci Nutr. 2026 Aug 2;14(8):e72199. doi: 10.1002/fsn3.72199 (PMC13429941; doi:10.1002/fsn3.72199)
Supplement: Supplementary file 6 — Table S2: Effect of applied factors on the study parameters during the storage. [file FSN3-14-e72199-s006.docx]

Supplementary Appendix **Table 2** Effect of applied factors on the study parameters during the storage

| **Parameter** | Study factors and their interactions | | | | | | |
| --- | --- | --- | --- | --- | --- | --- | --- |
|  | **SC** | **MO** | **T** | **MO**×**SC** | **SC**×**T** | **MO**×**T** | **MO**×**SC**×**T** |
| CFU, *Lactobacillus spp.* | 0.000 | 0.016 | 0.013 | NS | NS | NS | NS |
| CFU, *S. thermophilus* | 0.000 | 0.000 | NS | 0.000 | 0.000 | 0.001 | 0.000 |
| Lactic acid | NS | NS | 0.000 | NS | NS | NS | NS |
| pH | NS | NS | 0.000 | NS | NS | NS | NS |
| Total protein | NS | NS | NS | NS | NS | NS | NS |
| NPNC | NS | NS | 0.000 | NS | 0.089 | 0.006 | NS |
| Proteolytic activity | 0.046 | NS | 0.000 | NS | 0.004 | NS | NS |
| FAAs – Glu, Tyr, Sum of FAAs | 0.000 | NS | 0.000 | NS | 0.000 | NS | NS |
| FAAs – Ser, Gly, Pro, Arg | 0.000 | NS | NS | NS | 0.03-0.045 | NS | NS |
| FAAs – Asp, His, Lys, Val, Phe, Sum of EAAs | 0.000 | NS | NS | NS | NS | NS | NS |
| FAAs – Thr, Ala, Leu, Sum of EAA | NS | NS | NS | NS | NS | NS | NS |
| FAA – Met | 0.000 | 0.000 | 0.000 | 0.000 | 0.000 | 0.000 | 0.000 |
| FAAs – Sum of BCAAs, Ile | 0.000-0.003 | NS | NS | 0.000-0.036 | NS | NS | NS |
| PFs – Sum of PFs, α-LA, β-LG | NS | NS | 0.001-0.04 | NS | NS | NS | NS |
| PFs - β-CN, α-CN | NS | NS | NS | NS | NS | NS | NS |
| PF - κ-CN | 0.000 | NS | 0.000 |  |  |  |  |
| Abbreviations: MO – milk origin; SC – starter culture; T – sampling time; CFU – colony founding units; NPNC – non-protein nitrogen compounds; FAAs – free amino acids; PFs – protein fractions; NS – not significant (p > 0.05) | | | | | | | |
